# Consensus statement on novel glucose-related metrics obtained through advanced medical devices: English version

**DOI:** 10.1007/s13340-025-00820-2

**Published:** 2025-06-18

**Authors:** Rimei Nishimura, Yosuke Okada, Akio Kuroda, Junichi Suzuki, Yushi Hirota, Munehide Matsuhisa, Mizuki Ishiguro, Takayuki Ohno, Yuka Suganuma, Kenichi Tanaka, Atsuhito Tone, Akane Yamamoto, Sumiko Yoshida

**Affiliations:** 1 Division of Diabetes, Metabolism and Endocrinology, Department of Internal Medicine, Jikei University School of Medicine, 3-25-8 Nishi-shinbashi, Minato-ku, Tokyo, 105-8461 Japan; 2 First Department of Internal Medicine, University of Occupational and Environmental Health, Japan, 1-1 Iseigaoka, Yahatanishi-ku, Kitakyushu, Fukuoka 807-8555 Japan; 3 Diabetes Therapeutics and Research Center, Institute of Advanced Medical Sciences, Tokushima University, 3-18-15 Kuramoto-cho, Tokushima, Tokushima 770-8503 Japan; 4 Department of Pediatrics, Nihon University School of Medicine, 30-1 Oyaguchi-kami-cho, itabashi-ku, Tokyo, 173-8610 Japan; 5 Division of Diabetes and Endocrinology, Department of Internal Medicine, Kobe University Graduate School of Medicine, 7-5-1 Kusunoki-cho, Chuo-ku, Kobe, Hyogo 650-0017 Japan; 6 Department of Internal Medicine, Diabetes Center, Okayama Saiseikai General Hospital, 2-25 Kokutai-cho, Kita-ku, Okayama, Okayama 700-8511 Japan; 7 Department of Clinical Research, Shikoku Medical Center for Children and Adults, 2-1-1 Sen-yu-cho, Zentsuji, Kagawa 765-8507 Japan

**Keywords:** Continuous glucose monitoring, Glycemic excursion, Glycemic variability [GV], Glucose target, Glucose control goal, Time in range (TIR)

## Overview

### How should novel glycemic measures derived from continuous glucose monitoring (CGM), such as time in range (TIR), be applied in daily clinical practice for the management of diabetes mellitus?

Since the 1990 s when the Diabetes Control and Complications Trial (DCCT) [2], the United Kingdom Prospective Diabetes Study (UKPDS) [3] and the Kumamoto Study [4] demonstrated that achieving HbA1c less than 7% reduces the development and progression of microangiopathy in both type 1 and type 2 diabetes. As a result, HbA1c has become firmly established as the gold standard measure for the management of diabetes mellitus worldwide.

**Fig. 1:**
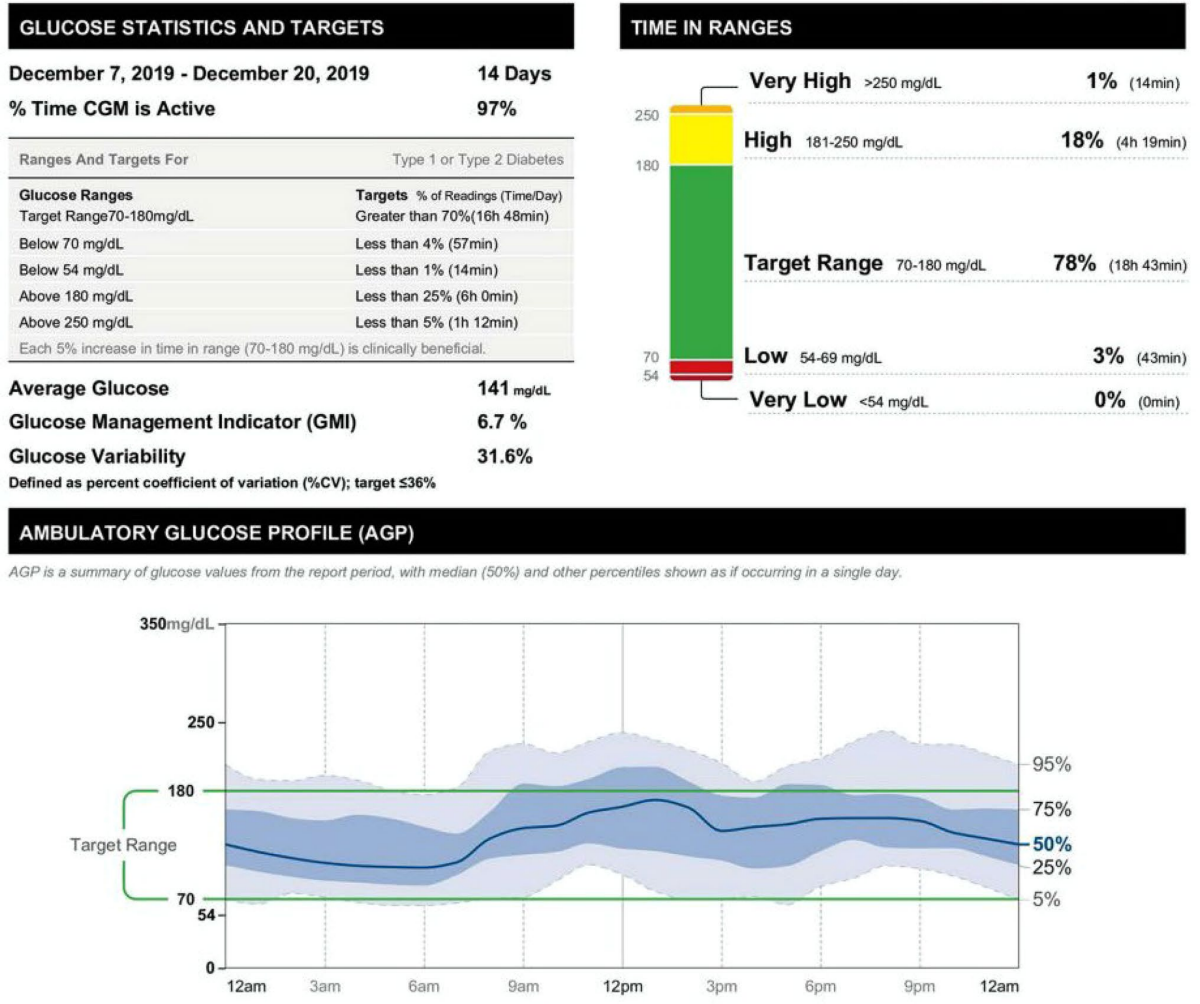
Example of a CGM-derived metric analysis report (excerpt displaying relevant metrics, e.g., proportion of time spent in the target glucose range [TIR], on the upper right of the diagram, and an ambulatory glucose profile [AGP] in the lower middle of the diagram)

However, since continuous glucose monitoring (CGM) devices became available and were covered by health insurance in Japan in 2010—and rapidly adopted worldwide, especially in Western countries—the International Conference on Advanced Technologies and Treatment for Diabetes (ATTD) introduced international consensus recommendations on “time in range” (TIR) as part of CGM-derived metrics for glycemic control status [5], which defined the glycemic target as ranging between 70 and 180 mg/dL, TIR (%) as proportion of time spent in this target range, “time above range” (TAR) (%) as proportion of time spent above the target range, and “time below range” (TBR) (%) as proportion of time spent below the target range, leading to TIR, TAR, TBR and ambulatory glucose profile (AGP) (to be discussed later) being included as default CGM-derived metrics for glycemic control status, to reflect the ATTD recommendations (Fig. 1).

Indeed, the use of TIR, TAR, and TBR helps formulate a highly relevant strategy for glycemic control in individuals with diabetes, i.e., one which consists in achieving their TIR target by decreasing their TAR, while at the same time minimizing their TBR to avoid hypoglycemia.

In real-world clinical settings, the use of these metrics has indeed not only made it possible to identify individuals with diabetes who are prone to hypoglycemia due to low HbA1c levels associated with a high proportion of TBR; it has also provided a point of reference in individualizing approaches to meet the varying needs of those failing to achieve their HbA1c target.

**Fig. 2:**
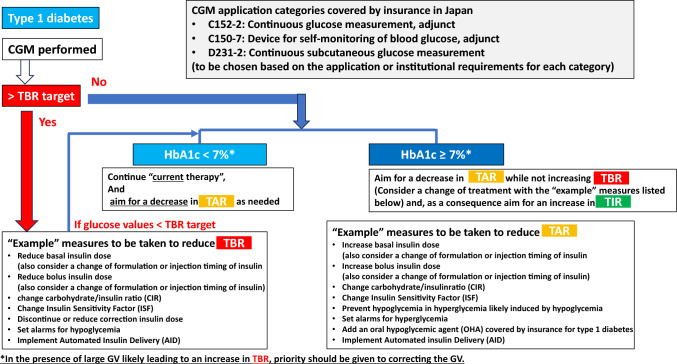
Policies for and responses to individuals with type 1 diabetes according to the proportions of time they spent within, below or above the target glucose range (i.e., time in range [TIR], time below range [TBR] and time above range [TAR]) GV, glycemic variability

**Fig. 3:**
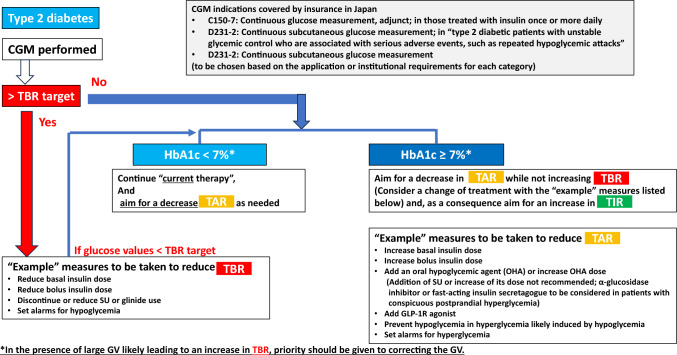
Policies for and responses to individuals with type 2 diabetes according to the proportions of time they spent within, below or above the target glucose range (i.e., time in range [TIR], time below range [TBR] and time above range [TAR])

This report provides guidance on interpreting and responding to TIR, TAR and TBR readings made available through CGM evaluations for individuals with type 1 diabetes and type 2 diabetes is provided as that summarized by the HbA1c target achieved in Figs. 2 and 3, respectively.

The overarching principle here is to make every effort to avoid any increase in TBR in individuals with type 1 and type 2 diabetes alike and regardless of their HbA1c values, with particular attention given to monitoring those failing to achieve the HbA1c target of less than 7% (or any appropriate target individually flexibly determined for them) to see if they have achieved their TBR target or suffered an increase in TBR subsequent to a treatment change. It is also recommended that treatment strategy be formulated for those with HbA1c values 7% or higher, based on the balance of their TIR, TAR and TBR and treatment options be chosen as required to decrease their TAR or to intensify treatment, thereby increasing their TIR (Figs. 2 and 3).

In addition, as a precaution, it is recommended that CGM devices be used in accordance with health insurance coverage categories and institutional requirements, as these are strictly defined, frequently updated, and vary by device.

In the current statement, therefore, a concrete exposition of these CGM metrics, their respective targets, as well as their supportive evidence, is provided to address relevant clinical questions in what follows.

It is hoped that the current consensus statement will contribute to the effective use of CGM-derived metrics, such as TIR, in clinical practice.

### Clinical question: What are the measures currently available for glycemic variability (GV)?

Measures for glycemic variability (GV) in individuals with diabetes (included in the ATTD international consensus on TIR) include: (1) standard deviations (SDs) of glucose values and (2) coefficients of variation (CVs) for glucose values. Of these, the SDs of glucose values are shown to be strongly positively correlated with mean glucose values, but their target SD values remain yet to be determined, while CVs for glucose values, calculated by dividing SDs of glucose values by mean glucose values, offer standardized values not influenced by increases or decreases in mean glucose values. Again, given that individuals with type 2 or type 1 diabetes receiving sulfonylurea (SU) or insulin therapy are known to be placed at significantly increased risk of hypoglycemia when the CVs for their glucose values are 36% or higher [6], the ATTD international consensus on TIR recommends that, for individuals with type 1 diabetes, the CVs for their glucose values be controlled at 36% or lower [7]. Of note, recent research reveals an interrelationship between an array of clinical markers, including those for GV and oxidative stress, suggesting that every effort should be made to minimize GV as far as possible in individuals with diabetes (Table 1)

**Table 1:** Major metrics for glycemic variability (GV)

Metric	Description
Standard deviation (SD)	SD of glucose values
Percent coefficient of variation (%CV)	Ratio of the SD to the mean, expressed as a percentage, indicating relative variability of a data set Formula: SD/mean glucose value × 100 Target in type 1 diabetes: 36% or lower
Mean amplitude of glucose excursions (MAGE)	Range of mean glucose values Mean of blood glucose values exceeding 1 SD from the 24‐h mean blood glucose
Mean of daily differences (MODD)	Indicator of day-to-day GV Average of the difference between values on different days but at the same time

Other metrics for GV in individuals with diabetes (not included in the ATTD international consensus on TIR) include: (3) mean amplitude of glycemic excursions (MAGE) calculated as a mean of glucose values exceeding 1 SD from the 24-h mean glucose derived from CGM, which offers the advantage of not being influenced by mean glucose values and is shown to be associated with cardiovascular events [8]; and 4) mean of daily differences (MODD) calculated as a mean of absolute glucose values measured at the same time point within a 24-h interval and used as a between-day GV index (Table 1).

## Main points of interest

SDs of glucose values are strongly positively correlated with mean glucose levels. While CVs for glucose, calculated by dividing the SD by the mean glucose value, provide standardized metrics independent of mean glucose levels, maintaining CVs at 36% or lower is recommended for individuals with type 1 diabetes from a safety perspective.

### Clinical question: What level of accuracy is required of CGM and what precautions are needed?

**Fig. 4:**
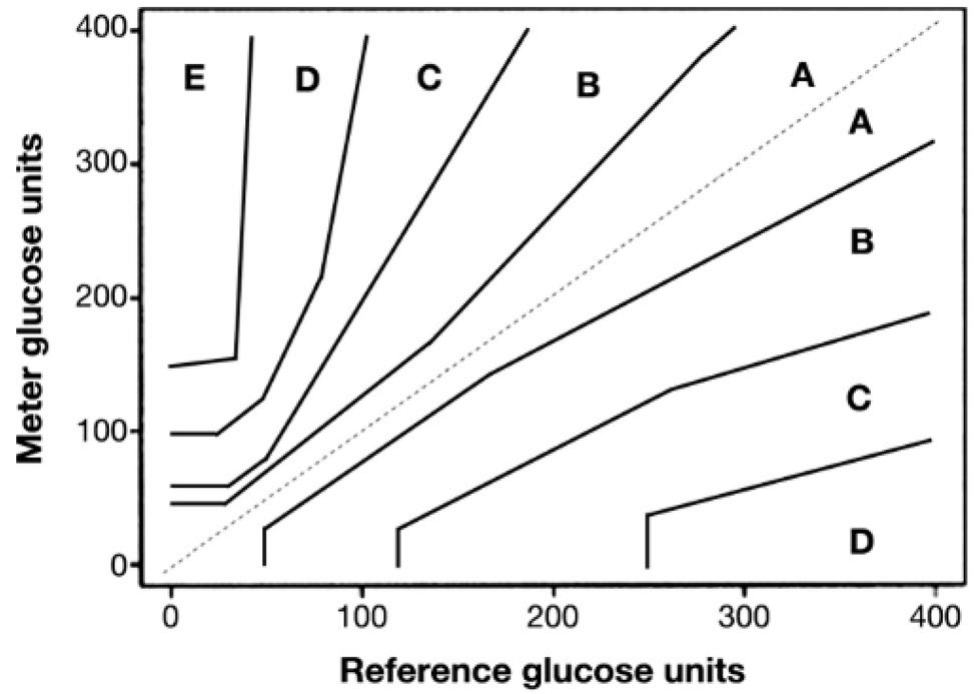
Parkes consensus error grid [10] (printed with permission from the *Journal of Diabetes Science and Technology*)

While the level of accuracy required of CGM is often presented as the mean absolute relative difference (MARD) (%) between the reference glucose values and the CGM readings with the MARD expected to range somewhere between 10 to 12% [9], to date, no clear target value for the MARD has been made available. The Parkes error grid is among the accuracy measures of interest, in that it allows medical devices to be evaluated for their clinical accuracy, demonstrating that 95% of all measured glucose values are supposed to fall within 15% of the reference value for glucose levels ≥ 100 mg/dL and within 100 mg/dL ± 15 mg/dL for glucose values < 100 mg/dL, with 99% of all measured glucose values falling within zones A and B in the Parkes consensus error grid [10] (Fig. 4).

It should be noted here that CGM devices do not display glucose values at the time of measurement but estimates as calculated based on measured glucose concentrations in hypodermal tissue at 1- to 5-min intervals according to their respective algorithms [11] and that these hypodermal concentrations represent values obtained 5–10 min earlier than those obtained at the time of measurement [12–14]. Thus, given that a discrepancy exists between the hypodermal glucose concentrations and actual glucose values due to the time lag and a lack of awareness of this could lead to the presence of hypoglycemia or excessive intake of supplementary food during hypoglycemia being overlooked, it is deemed desirable that measured glucose values are used together with CGM when it comes to evaluating individuals with diabetes for hypoglycemia or hyperglycemia.

## Main points of interest

- To date, no definitive target value for the accuracy required of CGM has been proposed or determined.
- The glucose values displayed on CGM devices are estimates derived from interstitial fluid measurements, reflecting readings taken 5–10 min prior to the displayed time.

### Clinical question: Is it necessary to use self-monitoring of blood glucose (SMBG) concurrently with CGM?

The management of diabetes mellitus aims at bringing glucose values as close to their target glucose values as possible through diet/exercise therapy, combined, as needed, with pharmacotherapy with oral hypoglycemic agents or insulin therapy.

As an integral part of the management of individuals with diabetes mellitus, CGM is intended to provide glucose concentrations as continuously measured in interstitial fluid using a glucose sensor inserted into hypodermal tissue, while self-monitoring of blood glucose (SMBG) is intended to provide glucose values as measured by a glucose meter using a drop of capillary blood from a needle’s pinprick on the patient’s finger. Given the discrepancy between CGM-measured and actual glucose values, it was previously required that glucose values available from SMBG performed one to four times daily be fed into the CGM device for calibration purposes. Furthermore, due to this discrepancy, it was also required that SMBG be used concurrently with CGM when evaluating individuals with diabetes for hypoglycemia or hyperglycemia, although, with this procedure in place, CGM devices caused few problems, offering sufficient level of accuracy for use in daily clinical practice.

Of note, all CGM devices made recently available for use in Japan are now equipped with an algorithm-based, self-calibrating capacity, thus obviating the need for SMBG-based calibration. It remains to be seen, however, if CGM devices could be deemed to stand alone without the aid of SMBG.

## Main points of interest

- With recent CGM devices, SMBG-based calibration is no longer required for their use.
- Further study is required to examine whether CGM devices may be deemed self-sufficient without the aid of SMBG.

### Clinical question: What are time in range (TIR) and time above range (TAR) as glycemic metrics?

**Fig. 5:**
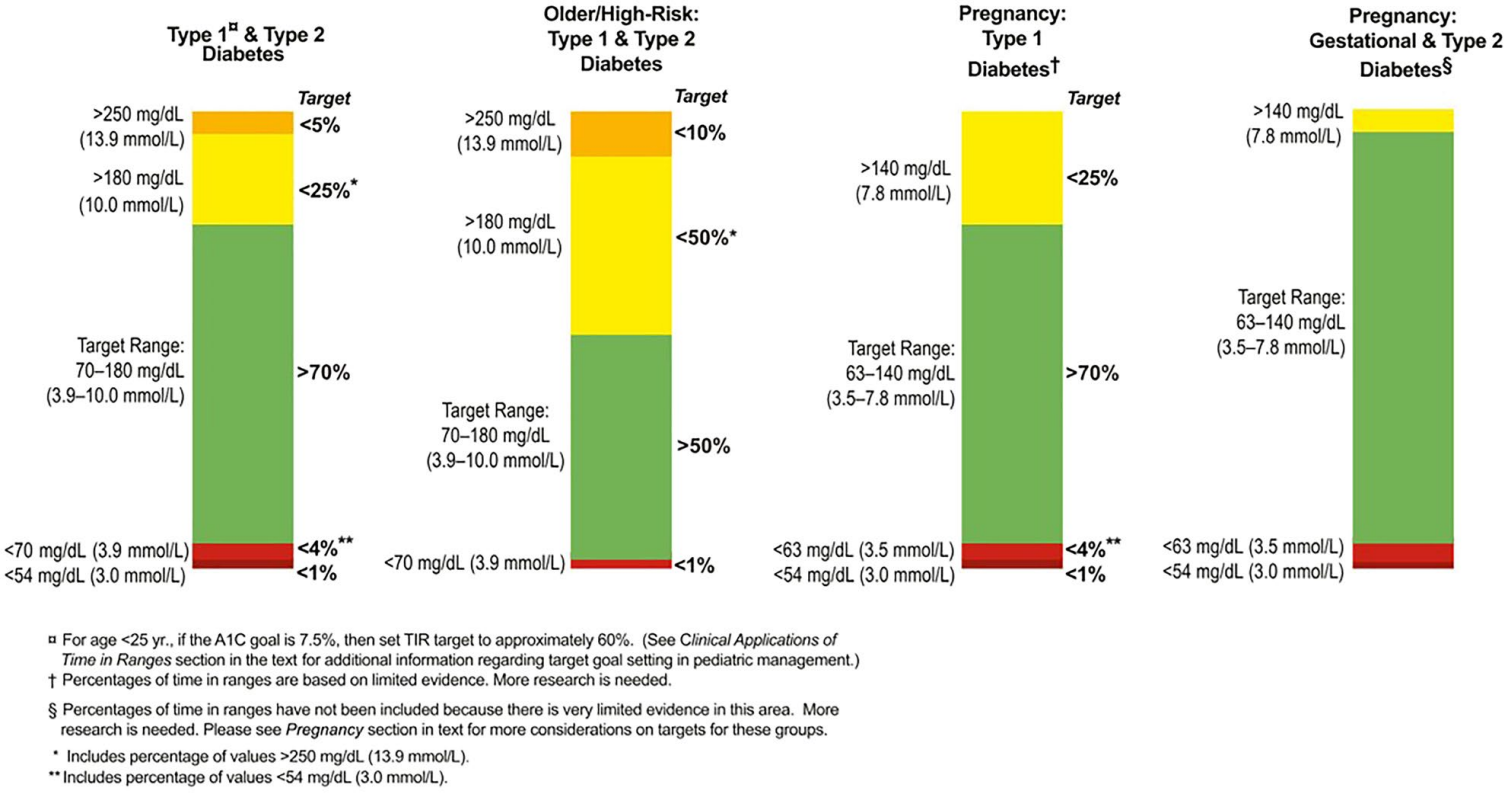
CGM-based targets for different diabetes populations [5]

As noted above, the ATTD set forth its consensus recommendations on time in range (TIR) in 2019 as part of the CGM-derived metrics for glycemic control status (Fig. 5) [5].Given that controlling postprandial glucose values to < 180 mg/dL is shown to reduce the onset of cardiovascular disease (CVD), such as microangiopathy and ischemic heart disease [15, 16], the ATTD consensus recommendations defined the TIR range as 70–180 mg/dL and the proportion (%) of time spent in this range as TIR, with TIR > 70% defined as the target in usual clinical practice for diabetes in light of the research finding suggesting that achieving a TIR of 70% or higher is consistent with achieving HbA1c 7% [17]; the consensus recommendations also defined the proportion (%) of time spent above the TIR as “time above range” as TAR, with TAR subcategorized into level 1 (glucose values 181–250 mg/dL) and level 2 (glucose values > 250 mg/dL), with the treatment target in usual clinical practice defined as TAR < 25% (glucose values > 180 mg/dL) and TAR < 5% (glucose values > 250 mg/dL), respectively.

## Main points of interest

- TIR refers to the proportion (%) of time spent in the glucose range between 70 and 180 mg/dL with TIR > 70% defined as the target in usual clinical practice, while TAR refers to the proportion (%) of time spent in glucose levels > 180 mg/dL, with TAR < 25% (glucose levels > 180 mg/dL) and TAR < 5% (glucose levels > 250 mg/dL) defined as the TAR targets in usual clinical practice.

Thus, it is essential to focus on increasing TIR as a measure of time spent in optimal glucose levels while at the same time decreasing TAR and TBR to achieve and maintain favorable glycemic control in individuals with diabetes safely and efficiently.

### Clinical question: What is the relationship between TIR and microangiopathy?

Reports on the relationship between TIR and microangiopathy are gradually increasing, leading to the publication of a systematic review on this topic in type 2 diabetes in 2022 (Table 2) [18]. This systematic review included 11 studies accounting for a total of 13,987 individuals for analysis, where 10 studies, including two in Japanese, were conducted in Asians, while four, four and seven studies assessed for retinopathy, nephropathy and neuropathy, respectively. Multiple studies have suggested that decreased TIR is associated with the severity of albuminuria and retinopathy, as well as with the prevalence of peripheral neuropathy and cardiac autonomic disorder. The caveat is, however, that not all studies have reproduced consistent results for this association and that only four studies were based on CGM data continuously collected over a 14-day period, i.e., the recommended duration for CGM data collection, with a majority of studies reporting results based on CGM data collected over a shorter period, thus calling for caution in interpreting the conclusions drawn. Again, the systematic review included not only studies reporting an association between TIR and microangiopathy even after adjusting for HbA1c values; it also included studies reporting to the same effect without adjusting for HbA1c values.

**Table 2:** Baseline characteristics of studies evaluating the association between CGM-derived TIR and microvascular complications among type 2 diabetes [18]

Characteristics	All included studies (*n* = 11)	TIR and diabetic retinopathy (*n* = 4)	TIR and diabetic nephropathy (*n* = 4)	TIR and diabetic neuropathy (*n* = 7)
Sample size, *n* (range)	466 (105–5901)	2315.5 (281–5901)	932.5 (281–5901)	349 (105–740)
Sex (%)				
Male	60.8	58.1	62.0	62.6
Female	39.2	41.9	38.0	37.4
Age, years, mean (SD)	59.3 (1.3)	62.1 (0.99)	61.6 (0.4)	59.1 (2.8)
Baseline A1c, %, mean (SD)	8.2 (0.5)	8.0 (0.6)	7.8 (0.4)	8.1 (0.6)
Duration of diabetes, years, mean (SD)	11.3 (1.0)	11.8 (0.7)	13.1 (0.2)	11.0 (1.1)
Study location (*n*)				
China	5			
South Korea	2			
Japan	2			
India	1			
USA	1			
CGM device used (*n*)				
Medtronic	5			
Abbott FreeStyle Libre	4			
Meiqi	1			
Medtronic + Meiqi	1			
Duration of CGM use (*n*)				
3 days	4			
14 days	4			
3 and 6 days for GOLD (Medtronic) and iPro2 (Medtronic), respectively^‡^	2			
Two 6-day periods, separated by 2 weeks	1			
CGM device calibrations (*n*)				
Not applicable	4			
At least two times per day	3			
At least four times per day	3			
Not reported	1			

Studies showing an association between TIR and microangiopathy in type 1 diabetes are even fewer, thus lacking in supportive evidence. Of interest here is, however, that the DCCT demonstrated that the hazard ratios for retinopathy and micro-albuminuria increase by 64% and 40% with every 10-point decrease in TIR as assessed by a 7-point SMBG profile [19]. Again, a study of 515 individuals with type 1 diabetes on sensor augmented pump (SAP) therapy demonstrated that those with microangiopathy had a lower TIR compared to those without (60.4% ± 12.2% vs. 63.9% ± 13.8%), suggesting that TIR is an independent risk factor for microangiopathy [20].

## Main points of interest

- Research evidence is gradually building that suggests the relationship between CGM-derived TIR and microangiopathy, but much of it is derived from retrospective, observational studies, thus highlighting the need for evidence from prospective studies.

### Clinical question: What is the relationship between TIR and macroangiopathy?

Evidence is currently building to support the relationship between TIR and macroangiopathy. A study of 152 individuals with type 1 diabetes without CVD [21] is of note, in that it showed that the presence of carotid artery plaques was not associated with TIR but with TBR alone (odds ratio [OR], 1.51 [1.07–2.13]), while, in contrast, a study of 2,215 individuals with type 2 diabetes evaluating an association between carotid intima-media thickness (CIMT) as a surrogate maker for CVD and TIR found that TIR was significantly decreased in those with abnormal CIMT (defined as mean CIMT ≥ 1.0 mm) (*P* < 0.001) and that the prevalence of CIMT tended to decrease with increasing TIR (*P* < 0.001) with the risk of having abnormal CIMT shown to decrease by 6.4% with a 10% increase in TIR, although these results were not adjusted for HbA1c [22].

Also of interest is a study that followed up a total of 515 individuals with type 1 diabetes receiving SAP for two years [20], which found that the risk factors for macroangiopathy in these individuals included age (OR, 1.08 [1.03–1.14]; *P* = 0.003) and HbA1c (OR, 1.80 [1.02–3.14]; *P* = 0.044), while TIR was not significantly associated with macroangiopathy. The caveat is, however, that the study may have suffered from its inadequate follow-up duration, given that macroangiopathy is likely to take longer than microangiopathy to observe develop.

In contrast, another study conducted in individuals with type 2 diabetes is more worthy of attention, in that it followed up as many as 6,225 individuals for as long as 6.9 years [23], which demonstrated that a total of 287 cardiovascular deaths occurred during follow-up and found, after adjustment for multiple factors (i.e., age, sex, smoking status, duration of diabetes, body mass index [BMI], systolic blood pressure [SBP], triglyceride/HDL-C/LDL-C levels, history of malignancy or CVD, and use of antihypertensives, aspirin and statins), that the hazard ratio (HR) for cardiovascular death was 1.85 (1.85 [1.25–2.72]; *P* = 0.015) among those with the lowest TIR (≤ 50%) suggesting a significant increase in cardiovascular death, compared to those with the highest TIR (> 85%).

As it is, there is still a paucity of studies assessing TIR and macroangiopathy for correlation and accumulation of evidence is required to delve further into this issue. At present, while some reports suggest a role for TIR in the onset of abnormal CIMT or increase in risk of cardiovascular death and hence a rationale for aiming for higher TIR to reduce the onset of macroangiopathy, accumulation of further relevant research findings is awaited.

## Main points of interest

- Accumulation of further evidence is required to determine the relationship between TIR and macroangiopathy, while some reports suggest a role for low TIR in the onset of abnormal CIMT or the increase in risk of cardiovascular mortality and hence a rationale for aiming for higher TIR to reduce the onset of macroangiopathy.

### Clinical question: What is the relationship between TIR and life prognosis in individuals with diabetes?

At present, no adequate evidence exists to determine the relationship between TIR and life prognosis in individuals with diabetes. Of potential interest here is the study of Lu et al. [23], which followed up a total of 6225 individuals with type 2 diabetes (mean age, 61.7 years) hospitalized during the 10 years between 2005 and 2015 until their death or 2018 and investigated the relationship between TIR and the increase in all-cause and CVD mortality using their 72-h CGM data following admission. The investigators found that, the HRs for all-cause mortality for those with TIR 71–85%, 50–70%, and 50% or lower were 1.23 (95% confidence interval [CI] 0.98–2.19), 1.30 (95% CI 1.04–1.63), and 1.83 (95% CI 1.48–2.28), respectively, compared to those with TIR > 85%, while the HRs for CVD mortality were 1.35 (95% CI 0.90–2.04), 1.47 (95% CI 0.99–2.19), and 1.85 (95% CI 1.25–2.72), respectively, and that TIR and all-cause mortality were inversely correlated, with the risk for all-cause and CVD mortality shown to increase by 1.08- and 1.05-fold, respectively, with every 10% decrease in TIR. However, some regard the study results as not readily generalizable, as they suffer not only from the CGM data used, which were collected over a short period of time and are now dated, but from unavailability of detailed data on the participants’ underlying diseases or history of insulin therapy [24].

Thus, further epidemiologic studies or randomized clinical studies are required to prove the correlation between TIR and life prognosis in individuals with diabetes.

## Main points of interest

- At present, studies investigating the relationship between TIR and life prognosis are very limited, with only one notable study reporting that a lower TIR is significantly associated with higher all-cause and cardiovascular disease (CVD) mortality.

### Clinical question: How is the TIR target to be defined for individuals with type 1 diabetes?

According to the 2019 ATTD consensus recommendations on TIR, the recommended TIR, TBR and TAR targets for individuals with type 1 and type 2 diabetes are defined as 70%, < 4% and < 25%, respectively, for individuals with type 1 and type 2 diabetes alike [5], with no different targets set for either population. Likewise, the Consensus Report published jointly in 2021 by the American Diabetes Association (ADA) and the European Association for the Study of Diabetes (EASD) followed suit, recommending the TIR target of > 70% as well as similar TBR and TAR targets to those recommended by the ATTD consensus [25].

In this context, studies evaluating TIR and HbA1c values for their correspondence are of interest as providing supportive evidence for the recommended TIR target. A meta-analysis of CGM data derived from four randomized trials accounting for a total of 545 adult individuals with type 1 diabetes showed not only that TIR values of 70% and 50% approximately corresponded to HbA1c values of 7.0% and 8.0%, respectively but that a 10% increase in TIR corresponded to a 0.5% decrease in HbA1c [17]. Likewise, another meta-analysis of 18 studies in individuals with type 1 and type 2 diabetes showed that a TIR value of about 65% corresponded to an HbA1c value of 7.0% and that a 10% change in absolute TIR value corresponded to a 0.8% change in HbA1c [26]. Thus, taken together, it is concluded that, while findings on the correspondence between TIR and HbA1c values vary among studies, a TIR value of 65–75% may be deemed to be consistent with an HbA1c value of 7.0% in adults [27].

Nonetheless, the fact remains that mean TIR values vary widely among individuals with type 1 diabetes depending on the treatment in place. Indeed, while the DIAMOND study in 158 individuals with type 1 diabetes receiving multiple daily insulin injections while on CGM reported a mean TIR of 51% in these individuals, studies of individuals with type 1 diabetes receiving hybrid closed loop (HCL) or advanced HCL technology-driven insulin pump therapy reported a TIR of 70–75% across the board [28–32], while TIR was not an indicator of hypoglycemia.

Thus, while a TIR target of > 70% is recommended for individuals with type 1 diabetes, as a rule, it should be noted that the TIR target may need to be individually determined in some cases, with consideration given to patient-related factors, such as the treatment in place and associated risk of hypoglycemia, particularly in elderly individuals or those at high risk of hypoglycemia, where the TIR target could be set to as low as > 50% [5].

## Main points of interest

- As a rule, the TIR target for individuals with type 1 diabetes is defined as > 70%.
- The TIR target may need to be individually determined in some cases, with consideration given to patient-related factors, such as the treatment in place and associated risk of hypoglycemia.

### Clinical question: How should the TIR target be determined for individuals with type 2 diabetes receiving insulin therapy?

The TIR targets currently recommended [5] are TIR > 70% for both type 1 and 2 diabetes with glucose levels 70–180 mg/dL and TIR > 50% for older and/or high-risk individuals [5]. High-risk individuals include those with hypoglycemia unawareness, a higher risk of complications, or prolonged insulin therapy [5], while, to date, no individualized TIR targets have been established based on specific diabetes treatments or the method of insulin therapy.

First, the REPLACE study reported a mean TIR of 56.7% and a mean TBR of 2.5% in type 2 diabetes treated with multiple daily insulin injections (MDI) or continuous subcutaneous insulin infusion (CSII) following intermittently scanned continuous glucose monitoring (isCGM) [33]. Also, the SHIFT study in Japanese individuals with type 2 diabetes also showed that their TIR improved from 62.5% at baseline to 69.5% following 90 days of isCGM, while their TBR remained unchanged (2.13% at baseline vs. 1.96% at the end of the study) [34]. The DIAMOND study reported a TIR of 61.3% and a TBR of 0.3% following real-time CGM in individuals with type 2 diabetes treated with MDI [35]. Furthermore, the MOBILE study reported a TIR of 59% and a TBR of 0.2% following real-time CGM in individuals with type 2 diabetes receiving insulin therapy [36]. Taken together, these findings suggest that individuals with type 2 diabetes receiving current insulin therapy can reliably achieve a TBR of < 4%, but achieving a TIR of > 70% is likely to remain challenging under current treatment strategies.

## Main points of interest

- The TIR target currently recommended are TIR > 50% for older or high-risk individuals with type 2 diabetes, including those with prolonged insulin therapy, and TIR > 70% for all other individuals with type 2 diabetes.

### Clinical question: How should the TIR target be determined for individuals with diabetes receiving insulin therapy in pregnancy?

It is ideal to ensure for individuals with diabetes in pregnancy that attention is focused at once on increasing their TIR and on reducing their TAR and glycemic fluctuations as rapidly and safely as possible.

It was reported in a study of women with type 1 diabetes in pregnancy that by controlling their glucose levels to the 63–140 mg/dL range using CGM during pregnancy, their neonatal outcomes significantly improved due to a decrease in maternal exposure to high glucose levels [37], which led the ATTD consensus recommendations on TIR to propose the TIR target of > 70% (glucose levels 63–140 mg/dL), the TAR target of < 25% (glucose levels > 140 mg/dL), and the TBR target of < 4% (glucose levels < 63 mg/dL) or < 1% (glucose levels < 54 mg/dL).

In contrast, there is a paucity of evidence to determine the targets for these CGM-derived metrics in individuals with gestational diabetes or type 2 diabetes in pregnancy, while the duration of exposure to high glucose levels is reported to be shorter at 33% in these individuals than in those with type 1 diabetes in pregnancy [38]. As a consequence, to date, no specific TIR, TAR or TBR targets have been recommended for individuals with gestational diabetes or type 2 diabetes in pregnancy in the ATTD consensus recommendations on TIR. Given the recent research highlighting the importance of setting more rigorous glycemic targets and ensuring nighttime glycemic control in gestational diabetes, however, accumulation of further research findings is awaited.

## Main points of interest

- For individuals with type 1 diabetes in pregnancy, their TIR should be controlled to the 63–140 mg/dL range, with their TIR, TAR and TBR targets set to TIR > 70% (glucose levels 63–140 mg/dL), TAR < 25% (glucose levels > 140 mg/dL), TBR < 4% (glucose levels < 63 mg/dL) or < 1% (glucose levels < 54 mg/dL). Likewise, the TIR should be construed as ranging between 63 and 140 mg/dL in type 2 diabetic women in pregnancy or women with gestational diabetes.

### Clinical question: How should the TIR target be determined for elderly individuals and individuals with advanced diabetic complications or hypoglycemia unawares who are being treated with insulin therapy?

In the ATTD consensus recommendations on TIR [5], it is recommended that for elderly individuals or individuals at high risk of diabetic complications, first and foremost, attention be focused on avoiding hyperglycemia while allowing hyperglycemia to last for a prolonged time, given their high risk of severe hypoglycemia due to hypoglycemia unawares, with the TIR, TBR and TAR targets set to > 50%, < 1%, and < 50%, respectively.

In elderly individuals, decreased adherence to their medications, aberrant hepatic metabolism due to decreased hepatic function, and polypharmacy lead to an increased risk of hypoglycemia [39]. While attention to these characteristics of elderly individuals led to the International Diabetes Federation (IDF) and the Japan Diabetes Society (JDS) recommending that the HbA1c target be individually determined for elderly individuals with diabetes with consideration given to their frailty or physical function, the ATTD consensus recommendations on TIR recommended a consistent HbA1c target across all age groups. Also, there is an argument against the use of TIR > 50% as the target for elderly individuals, given that it corresponds to HbA1c < 8.3% and thus is too low a target for those whose activities of daily living (ADL) remain intact [40]. Indeed, individuals with type 1 diabetes without frailty 60 years old or older receiving SAP therapy were shown to have a median TIR of 71% in one study [41], which also suggested that the TIR target may be set to higher than > 50% if hypoglycemia could be effectively avoided, given the TBR of 2% in these individuals.

Furthermore, the risk of hypoglycemia is shown to increase in individuals with advanced diabetic complications. Indeed, research findings are available that support the link between the severity of chronic kidney disease (CKD) stage and the incidence of hypoglycemia [42]. Again, as hypoglycemia occurs repeatedly, it leads to a rise in the threshold for counteractions against it, thus making it manifest as hypoglycemia unawares [43]. Among individuals at risk of hypoglycemia unawares, the risk of severe hypoglycemia (i.e., glucose levels < 54 mg/dL and hypoglycemia requiring the assistance of others for recovery [44]) is shown to be increased by sixfold in individuals with type 1 diabetes [45] and by 17-fold in individuals with type 2 diabetes on insulin therapy [46]. It is desirable that every effort is made to avoid severe hypoglycemia, given that it is shown to lead not only to a decrease in cognitive function [47] and QOL [48] but also to an increase in the risk for traffic accidents [49] or death [50]. Again, of all pharmaceutical agents responsible for severe hypoglycemia, insulin is shown to be foremost, accounting for 60.6% of all cases of severe hypoglycemia [51], and particular attention is required to watch for severe hypoglycemia in individuals receiving insulin therapy.

Thus, taken together, it is recommended that the TIR target be determined individually in elderly individuals or those with advanced diabetic complications with a focus on keeping the TBR to a minimum, with the TIR target of > 50% as a guide.

## Main points of interest

- In elderly individuals or those with advanced diabetic complications, it is recommended that the TIR target be determined individually with a focus on keeping the TBR to a minimum, with the TIR target of > 50% as a guide.

### Clinical question: How should the TIR target be determined for pediatric individuals with diabetes (pediatric insulin users)?

The International Society of Pediatric and Adolescent Diabetes (ISPAD) Clinical Practice Consensus Guidelines 2022 recommended the TIR target of > 70% with the TIR range defined as 70–180 mg/dL [52].

The mean TIR is reported to be about 50% in pediatric individuals whose exercise or eating habits are unstable compared to those in adult individuals with diabetes [53, 54]. An isCGM-based study of Japanese children with type 1 diabetes 3–18 years old also showed their mean TIR to be 50.7% in agreement with the above overseas studies, despite showing their mean TAR and TBR to be 37.5% and 11.8%, respectively, considerably higher values than those recommended in the ISPAD Guidelines [55]. Of note, an examination of these children for correspondence between HbA1c and TIR values revealed that HbA1c 7.0% corresponded to the TIR of 55.1%, while the TIR of > 70% corresponded to HbA1c 6.0%, suggesting that highly rigorous glycemic control is required to achieve the TIR target of > 70% [55].

Thus, taken together, it appears difficult to reduce glycemic fluctuations thereby achieving the TIR target of > 70% in pediatric individuals associated with unstable exercise and eating habits. Thus, it is desirable that the TIR target be determined individually in pediatric individuals using CGM with consideration given to their age and lifestyle.

## Main points of interest

- The targets for the CGM-derived metrics are similarly defined for pediatric individuals with diabetes to those in adults, with the TIR range defined as 70–180 mg/dL and the recommended TIR target defined as > 70%. However, this TIR target is often difficult to achieve in pediatric individuals, likely leading to an increase in their TBR. Thus, it is desirable that the TIR target be determined individually, considering such factors as their age, lifestyle and the current treatment regimen.

### Clinical question: how should the TIR target be determined for individuals with diabetes not receiving insulin therapy (including those with type 2 diabetes, elderly individuals with type 2 diabetes, and individuals with diabetes in pregnancy) be determined?

There is a paucity of evidence to draw on in determining the TIR target for individuals not receiving insulin therapy. Furthermore, at present, CGM is indicated by insurance in very few individuals with type 2 diabetes, elderly individuals with type 2 diabetes or individuals with diabetes in pregnancy who are not receiving insulin therapy and only indicated in “individuals with type 2 diabetes with unstable glycemic control who are associated with serious adverse events, such as repeated hypoglycemic attacks” (indication D231: subcutaneous continuous glucose monitoring).

However, CGM is expected to be increasingly performed in these individuals at their own expense. Thus, when it comes to performing CGM in these individuals, it is deemed desirable that the same targets be temporarily employed as those recommended for insulin users until further evidence becomes available to suggest otherwise, i.e., the TIR target of > 70%, > 50%, and > 70% (glucose values 63–140 mg/dL) in individuals with type 2 diabetes, elderly individuals with type 2 diabetes, and individuals with diabetes in pregnancy, respectively. Nonetheless, what applies for all individuals is the overarching principle that every effort should be made to reduce TBR as close to zero as possible. It goes without saying, of course, that this principle requires no supportive evidence.

## Main points of interest

- There is a paucity of evidence to draw on in determining the TIR target for individuals not receiving insulin therapy. However, it is deemed desirable that the same targets be temporarily employed as those recommended for insulin users, i.e., the TIR target of > 70%, > 50%, and > 70% (with the glucose values ranging from 63 to 140 mg/dL) in individuals with type 2 diabetes, elderly individuals with type 2 diabetes, and individuals with diabetes in pregnancy, respectively. What applies for all individuals, however, is the principle that TBR should not be increased but rather must be reduced as close to zero as possible.

### Clinical question: What is time in tight range (TITR) like as a glycemic metric? How should it be used in daily clinical practice?

In recent years, the advent of insulin pumps designed to automatically adjust basal insulin doses in response to CGM readings, as well as advances in pharmacotherapy for diabetes mellitus, has resulted in dramatic improvements in glycemic control in some individuals and even led to “diabetic remission” being proposed as a novel concept in some quarters.

In response to this move, the ATTD consensus recommendations have come to include the “time in tight range” (TITR) as a novel glycemic metric far more rigorous than TIR in 2023, where the TITR target is defined as the proportion (%) of time spent in the glucose range between 70 to 140 mg/dL.

The reason for defining the upper limit of TITR as 140 mg/dL is given below. A postprandial glucose level of 140 mg/dL or higher is defined by the IDF as postprandial hyperglycemia [56]. Also, in the 75-g oral glucose tolerance test (OGTT), a 2-h post-load plasma glucose level of 140 mg/dL or higher is defined as impaired glucose tolerance (IGT) [57]. Furthermore, postprandial hyperglycemia and IGT represent risk factors for the onset of CVD or death [58–60].

**Fig. 6:**
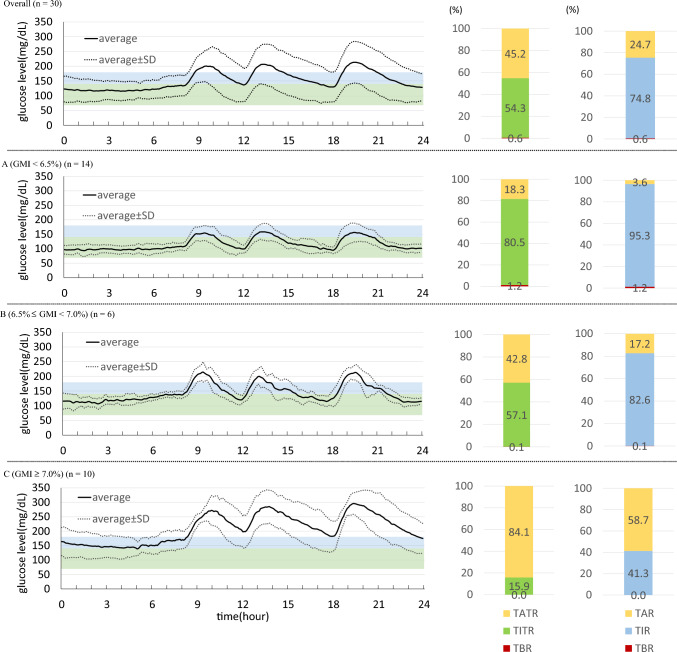
Comparison of CGM-derived data as stratified by GMI. TITR and TIR are shown as light green and light blue, respectively. All values are shown as Averages. *CGM* continuous glucose monitoring, *GMI* glucose management indicator, *TAR* time above range (glucose, > 180 mg/dL), *TATR* time above tight range (glucose, > 140 mg/dL), *TBR* time below range (glucose, < 70 mg/dL), *TIR* time in range (glucose, ≥ 70 mg/dL/≤ 180 mg/dL), *TITR* time in tight range (glucose, ≥ 70 mg/dL/≤ 140 mg/dL)

Attempts at narrowing the range of GV with TITR as a guide are thought likely to lead not only to quality glycemic control but to prevention of diabetic complications. Currently, several studies are available on the TITR target. First and foremost, a study of individuals with type 1 diabetes receiving insulin pump therapy is of interest, in that it led to the TITR target being proposed as TITR < 50% in these individuals [61]. Second, another study of Japanese individuals with type 2 diabetes is of note, as it led to the TITR target being estimated by glucose management index (GMI) value [62], where, when aiming for GMI values < 6.5%, the TIR target was estimated at about 100% but the TITR target was estimated at about 80% in these individuals (Fig. 6; left A), while, when aiming for GMI values 6.5–7.0%, the TIR target was estimated at about 80% and the TITR target was estimated at about 60% (Fig. 6; left B).

Therefore, expectations are mounting for the day when we will have the TITR target clearly defined for complete inhibition of diabetic complications.

## Main points of interest

- The preferential use of TITR in clinical practice is thought likely to lead to better glycemic control resulting in a greater reduction in the risk for diabetic complications than the use of TIR. In addition, TITR may have the potential to become a hallmark measure of “diabetic remission”. It is expected with interest that the TITR target will soon be defined with the accumulation of relevant evidence.

### Clinical question: What is “time below range” (TBR) like as a glycemic metric?

According to the ATTD consensus recommendations on TIR [5], “time below range” (TBR) is defined as the proportion (%) of time spent in the lower glycemic range than that for TIR and classified into level 1 (glucose levels 54–69 mg/dL) and level 2 (glucose levels < 54 mg/dL), with these criteria made consistent with those proposed by the International Hypoglycemia Study Group [43]. Of the levels of TBR thus defined, level 1 represents a clinically important range where it is necessary to implement measures to prevent further declines in blood glucose, irrespective of the presence or absence of hypoglycemic symptoms, while level 2 represents a range where prompt carbohydrate supplementation and measures to prevent hypoglycemia from becoming serious are required.

### Clinical question: How should the TBR target be determined?

TBR is shown to be less well correlated with TIR than TAR [26]. Thus, the goal of blood glucose management in individuals is to maintain TBR at level 1 and level 2 as low as possible to prevent severe hypoglycemia, rather than aiming to reduce TBR solely to achieve TIR targets.

Specifically, in usual clinical practice for individuals with type 1 and type 2 diabetes, attention should be focused on achieving the target for TBR (Level 1) is less than 4% (i.e., 1 h), and the target for TBR (Level 2) is less than 1% (i.e., 15 min) [4].

In older individuals or individuals with advanced diabetic complications who are both at high risk of hypoglycemia, particularly, every effort should be made to prevent hypoglycemia as much as possible, with a target of maintaining TBR (level 1) below 5 min and avoiding TBR (level 2) [4].

The goal of glycemic control in pregnant women with diabetes or gestational diabetes has been defined as consisting in aiming for the glucose range from 63 to 140 mg/dL. Thus, every effort should be made to achieve TBR in the range of 54–62 mg/dL less than 4%, and TBR below 54 mg/dL less than 1%, respectively. The caveat is, however, that as the rationale for these TBR targets was derived from that for the TIR targets, TBR targets should be appropriately determined based on a detailed analysis of the risk for severe hypoglycemia in these individuals.

## Main points of interest

- In aiming for a TIR of > 70%, it is preferable to aim for TBR < 4% and < 1% in level 1 TBR (glucose levels < 70 mg/dL) and level 2 TBR (< 54 mg/dL), respectively.
- In aiming for a TIR of > 50% in older individuals and individuals with advanced diabetic complications who are both at high risk of hypoglycemia, it is preferable to aim for TBR < 1% and 0% in level 1 TBR and level 2 TBR, respectively.

### Clinical question: What is the ambulatory glucose profile (AGP) as a glycemic metric, and how should it be used in daily clinical practice?

The ATTD consensus recommendations proposed that one-page summary reports called ambulatory glucose profiles (AGPs), i.e., reports so standardized as to allow for comparisons and evaluations over time, be used to visually capture changes and trends in GV over the course of time covered to provide insights. This AGP report consists of three sections (upper row, glucose-related statistics and relevant targets, such as TIR, GMI, % valid sensor duration; middle row, AGP graphs; and lower row, daily glucose profiles).

**Fig. 7:**
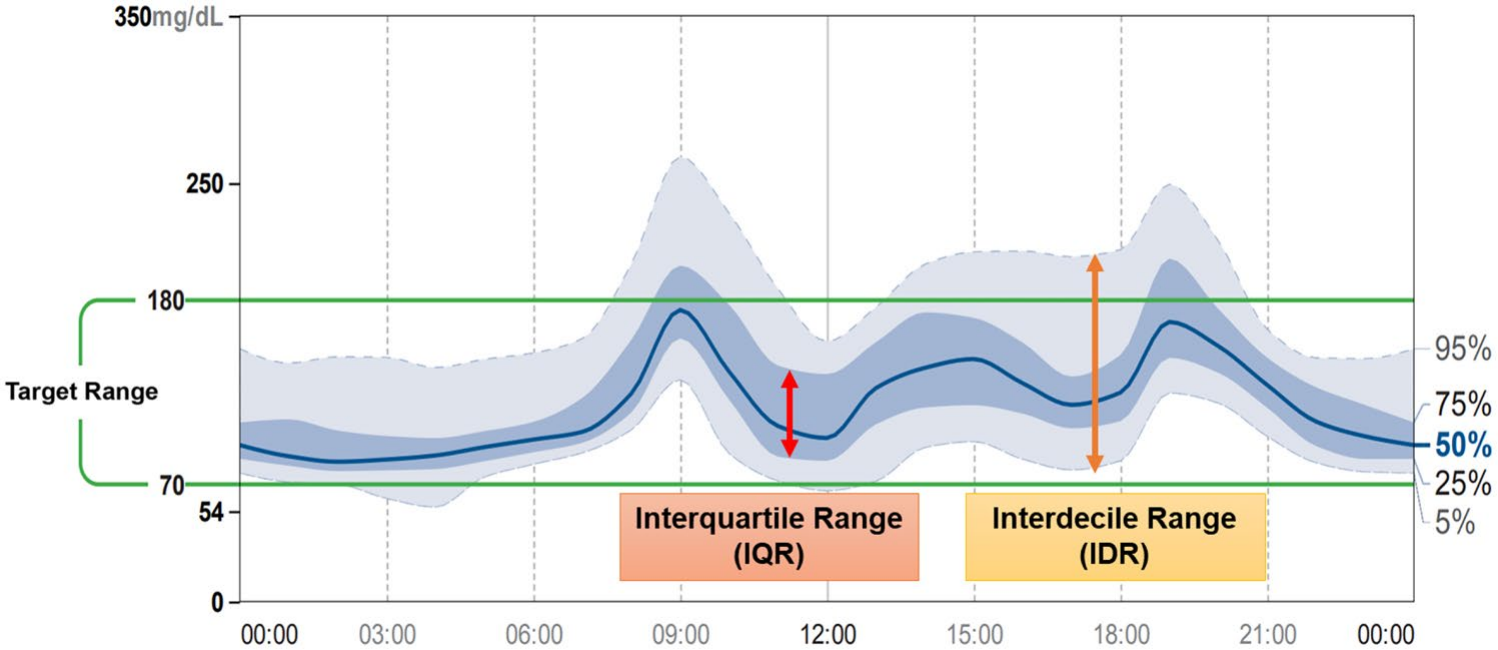
An example of AGP (ambulatory glucose profile)

Each AGP report features five different curves, with the median [50 percentile] glucose curve displayed in the middle, with the interquartile (25–75 percentile) range (IQR) glucose curves shown above and below the median curve, and with the interdecile (5–95 percentile) range (IDR) glucose curves provided further out and above and below the IQR curves (Fig. 7). In this diagram, daily GV is assessed with attention focused on upward/downward changes in the median glucose curve to identify the timeframe associated with the greatest changes in daily GV. Again, day-to-day GV is assessed with attention focused on day-to-day changes in the vertical width of the IQR or IDR glucose curves to identify timeframes associated with conspicuous GV. Thus, AGP is an analytical tool designed to allow for an easy visual grasp of timeframes in which hypoglycemia, hyperglycemia or conspicuous GV are likely to occur, thereby providing insight into changes or trends in GV over time.

In daily clinical practice for diabetes, AGP reports should be analyzed by following the steps detailed in Table 3. First, check to see if the CGM device shows a ≥ 70% valid sensor duration. Again, while CGM-derived AGP reports are usually intended to provide glucose profiles over a 14-day span as a default, longer monitoring periods should be considered if the CGM device displays a < 70% valid sensor duration or where hypoglycemia is an issue of interest [63, 64]. Next, focus on identifying patterns of hypoglycemia (considering frequency, duration, severity, and timeframes) and making necessary adjustments to avoid hypoglycemia. In assessing individuals for GV, attention should be given to the width of upward/downward changes in the IQR glucose curves (which are assumed to be influenced by treatment-related factors), as well as in the IDR glucose curves (which are assumed to be influenced by behavioral or lifestyle factors [63], where it is desirable that these vertical widths be kept as narrow as possible. It is also preferable that the % coefficient of variation (%CV) displayed on the upper row of each AGP report be controlled to 36% or lower [65]. In assessing glucose profiles for stability, focus on peak values for upward/downward changes, as well as their gradients, in the median glucose curve to ensure that every effort is made to keep the course of these values as flat as possible, while noting that these peaks may be found to cancel each other out, thus making the curve seem flat in individuals whose day-to day eating times and lifestyle patterns are shown to vary.

**Table 3:** Steps in the analysis of AGP reports

Step	Instruction	Note
Step 1	Examine data quality and TIR values	Check for ≥ 70% valid sensor duration, as well as duration of data coverage
Step 2	Identify patterns of hypoglycemia present	Check hypoglycemia for its frequency, duration and severity, as well as for timeframes associated with it
Step 3	Assess for GV	Check for IQR, IDR, and ≤ 36%CV
Step 4	Assess for glucose stability	Check for the median glucose curve (e.g., up/down peaks, their course and gradients)

*AGP* ambulatory glucose profile, *CV* coefficient of variation, *GV* glycemic variability, *IDR* interdecile range, *IQR* interquartile range

Thus, AGP is useful as it allows for a visual understanding of glycemic variability (GV), enabling the formulation and proposal of treatment plans focused on specific timeframes. It should also be noted that adequate discussions with individuals, including those of their lifestyle and treatment involvement, are part and parcel of decision-making regarding treatment or any change of treatment. In summary, concurrent use of various glycemic metrics such as TIR as well as graphs provided by AGP reports should help in the formulation of on-target treatment strategy for individuals with diabetes.

## Main points of interest

- Each AGP report features five different curves, with the median (50 percentile) glucose curve displayed in the middle, with the interquartile (25–75 percentile) range glucose curves shown above and below the median curve, and with the interdecile (5–95 percentile) range glucose curves provided further out and above and below the IQR curves.
- AGP provides a visual overview of the timeframes associated with hypoglycemia, hyperglycemia, daily GV, and day-to-day GV.
- AGP-based assessments, performed according to the steps described herein and with patient involvement, should lead to formulation and implementation of on-target treatment.

### Clinical question: What is Glucose Management Indicator (GMI) like as a glycemic metric?

Glucose Management Indicator (GMI) is a CGM-derived metric providing estimates of HbA1c values calculated using mean sensor glucose values, which are shown to closely resemble actual measured HbA1c values. Before 2018, this metric used to be referred to as “estimated A1c (eA1c)”; however, the new term “GMI” has been proposed for this metric to avoid confusion with actual laboratory-measured HbA1c values [66]. In addition, a reexamination of the formula used for this calculation led to a revised formula being suggested: GMI (%) = 3.31 + 0.02392 × mean sensor glucose value (mg/dL) [66]. It should be noted that as a rule, an increase of 25 mg/dL in mean sensor glucose value is deemed to correspond to a 0.6% increase in GMI, while mean sensor glucose values of 150, 175 and 200 mg/dL are deemed to correspond to a GMI of 6.9%, 7.5%, and 8.1%, respectively. While the International Consensus on Use of Continuous Glucose Monitoring (2017) [7] recommended that sufficient CGM-derived data, i.e., 70% or more of CGM data available over a 14-day period or those available over a course of 10 days or longer, be used to provide appropriate estimates of eA1c values, likewise, it is recommended that GMI values be calculated using similar CGM-derived data to those used in estimating eA1c values [66]. Of note, the ATTD consensus recommendations on TIR (2019) included GMI among the 10 most common metrics of interest in CGM-based assessments [5], and this has led to GMI being included as a metric in the analytical software used in all CGM devices currently commercially available in Japan.

## Main points of interest

- GMI represents a metric providing estimates of HbA1c calculated using mean sensor glucose value. For accurate estimates, it is recommended that at least 70% of CGM data collected over a 14-day period be used.

### Clinical question: How should GMI be used in daily clinical practice for diabetes mellitus?

The advantage of GMI over HbA1c most worth stressing is that it allows for shorter term assessments of glycemic control. Indeed, while actual measured HbA1c values each represent the proportion of glycated hemoglobin resulting from binding of glucose to hemoglobin, only reflecting changes in blood glucose over the past 1–2 months, GMI offers estimates of glucose values derived from daily mean sensor glucose values, thus allowing glycemic control status to be examined at time points closer to those of actual measurement.

The use of GMI may be of particular interest in situations requiring a timely grasp of glycemic control status, such as where individuals are expected to experience drastic worsening of glycemic control status during sick days or following steroid use or where they are expected to experience drastic improvements in glycemic control status due to initiation of diet therapy or intensification of exercise therapy.

Particularly in individuals in whom a discrepancy is likely to occur between their HbA1c and mean glucose values, e.g., those with acutely improving diabetes, acute-onset/worsening diabetes, iron deficiency, convalescing iron deficiency anemia, hemolysis, liver cirrhosis, dialysis, transfusions, or abnormal hemoglobinemia [67], GMI values are likely to reflect glycemic control status more accurately than HbA1c values.

One precaution to be noted in the use of GMI is that there is a discrepancy between laboratory-measured HbA1c values and GMI values, which, however, is reported to remain constant within the same individual [68]. Differences in this discrepancy between individuals are assumed to result from differences in their red cell lifespan [69] as well as in their glucose transfer through red cell membranes [70]. Again, in today’s digital landscape where CGM-derived data are increasingly managed by server-based clouds, such system has now allowed for the use of GMI values as an alternative to actual measured HbA1c values in remote clinical practice (i.e., virtual healthcare settings).

## Main points of interest

GMI enables short-term assessments of glycemic control and is especially useful for evaluating individuals whose glycemic control status is expected to change in the short term or whose condition may not accurately reflect laboratory-measured HbA1c values.

### Clinical question: In which situations is a discrepancy likely to occur between GMI and HbA1c values?

In the primary paper proposing the use of GMI [66], it is reported that, between GMI and HbA1c values, discrepancies of 0.3% or higher and 0.5% or higher are noted in 51% and 28% of individuals, respectively. Again, given that this discrepancy remains constant within the same individual, however, care should be taken to ensure that the HbA1c target is not lowered too much in individuals whose GMI values remain lower than their HbA1c values, while, conversely, the HbA1c target is not raised too high in individuals whose GMI values remain higher than their HbA1c values [66]. In addition, while the difference between GMI and HbA1c values (HbA1c–GMI) is often expressed as the hemoglobin glycation index (HGI), it should be noted that high HGI values are shown not only to be associated with diabetic complications [71] and CVD [72] but to vary from race to race [73]. While, as noted, differences in this discrepancy between individuals are assumed to result from differences in their red cell lifespan [69] as well as in their glucose transfer through red cell membranes [70], and an attempt has been made to provide more accurate CGM-based estimates of HbA1 values using a kinetic model [74], at present, no established methodology is available for estimating HbA1c values.

Where a discrepancy is noted between HbA1c and GMI values, therefore, consideration should first be given to potential issues in CGM sensor accuracy to assess for any discrepancy between glucose values derived from SMBG and those derived from blood sampling. It should also be noted that acute increases or decreases in difference between HbA1c and GMI values may be suggestive of changes occurring in glycemic control status over the short term. As individuals with conditions resulting in a discrepancy between their HbA1c and mean glucose values, as noted earlier, may also be found to be associated with a discrepancy between their GMI and HbA1c values, these individuals need to be assessed for the presence of any such condition. In addition, while the use of 70% or more of CGM-derived data available over a 14-day period is recommended for use in calculating GMI values [66], it should also be noted that GMI values calculated using CGM-derived data obtained over too short a period may not accurately reflect actual glycemic control status.

## Main points of interest

- While GMI and HbA1c values are shown to vary to a certain extent in some individuals, this discrepancy is shown to remain constant within the same individual. It should also be noted that acute increases or decreases in difference between HbA1c and GMI values may suggest changes occurring in glycemic control status over the short term. Consideration should also be given to potential issues in CGM sensor accuracy.
